# Parasocial interaction and problematic use of short-form video applications: unveiling the mediating mechanism

**DOI:** 10.3389/fpsyg.2025.1584685

**Published:** 2025-07-14

**Authors:** Qing Huang, Sihan Lei, Zhuo Chen

**Affiliations:** ^1^College of Media and International Culture, Zhejiang University, Hangzhou, China; ^2^International Communication Institute, Zhejiang University, Hangzhou, China; ^3^Department of Communications and New Media, National University of Singapore, Singapore, Singapore; ^4^Department of Financial Media, Peking University HSBC Business School, Shenzhen, China

**Keywords:** parasocial interaction, flow, fear of missing out, problematic use of short-form video applications, mediation effect

## Abstract

**Introduction:**

Problematic use of short-form video applications (SVA) has posed significant challenges to individuals' wellbeing in recent years. This study examines how parasocial interaction-a one-sided and imagined emotional engagement with vloggers—contributes to problematic SVA use.

**Methods:**

Based on the socio-psychological perspective and informed by the positive reinforcement and compensatory use approaches, the research explores how flow experience and fear of missing out mediate this association. An online survey was conducted among SVA users (N = 407). The collected dataset was analyzed using SPSS PROCESS Macro model 6 to test a serial mediation model.

**Results:**

Results support the mediation model, indicating that the reinforcement of positive feelings and the compensatory motivation for alleviating negative emotions jointly lead to the problematic behaviors.

**Discussion:**

The findings offer valuable insights into the socio-psychological processes underlying problematic SVA use and suggest potential intervention strategies to promote healthy usage of SVA.

## 1 Introduction

In recent years, short-form video applications (SVA), such as TikTok and Kuaishou, have become popular around the world, raising concerns about problematic use and its adverse impacts. As a form of interactive media, SVA enable users to share their experiences or watch others' everyday lives through 30- to 60-s videos (Feng et al., 2024). However, the immersive pleasure provided by watching short-form videos often leads users to engage in excessive interactions with SVA (Ye et al., 2022) and develop problematic use behaviors. Problematic SVA use describes an individual's compulsive behavior of using SVA, accompanied by sleep deprivation, emotional depression, and poor time management if SVA are inaccessible (Mu et al., 2022; Zhang et al., 2023).

The majority of prior studies considered problematic use of mobile applications a psychological issue and used a psychological perspective to explicate such behaviors. This approach investigates how positive reinforcement (i.e., reinforcing positive experience) and compensatory use (i.e., regulating negative emotions) lead to users' problematic behaviors (Yao et al., 2023; Yang et al., 2022; Zhang and Rau, 2021). Moreover, a limited number of studies adopted the interactive approach and demonstrated that user interaction with the application interface might contribute to the development of problematic use behaviors (Cao et al., 2025; Zhang et al., 2019). Noticeably, the (para)social interaction between users and vloggers is a common activity and experience in the context of SVA usage, which has been underexamined in the extant literature. Vloggers are content creators who produce vlogs (i.e., video blogs) based on their everyday lives and upload them to SVA platforms to attract followers. Many vloggers also perform in their videos (Gholamhosseinzadeh, 2023).^1^ In most cases, users interact with vloggers through watching, liking, commenting on, or sharing the videos created by their favorite vloggers, whereas vloggers seldom respond to user behaviors. These mediated behaviors lead users to establish a one-way connection with vloggers while imagining themselves engaging in a reciprocal relationship. This process is defined as parasocial interaction (Garg and Bakshi, 2024). Given that the interaction between users and vloggers tends to evoke users' psychological responses (Hsu, 2020), parasocial interaction may serve as an antecedent of positive reinforcement and compensatory use.

With such consideration, this study includes flow and fear of missing out, two psychological variables as mediators between parasocial interaction and problematic SVA use. The mediating role of flow represents the positive reinforcement approach to examining problematic use behaviors (Qin et al., 2022), while the mediating role of fear of missing out reflects the compensatory use approach (Mouakket and Aboelmaged, 2023; Wegmann and Brand, 2019). The literature review section will illustrate these two approaches and elaborate on the mediating roles of flow and fear of missing out accordingly.

By proposing a mediation model that addresses the associations between parasocial interaction, flow, fear of missing out, and problematic SVA use, this study aims to accomplish three goals: (1) to conceptualize users' parasocial interactions with vloggers as a common type of socio-psychological process in the setting of SVA usage; (2) to explicate the socio-psychological perspective in problematic SVA use research by introducing parasocial interaction and examining the mediating roles of flow and fear of missing out; and (3) to provide policy makers, platform corporations, and individuals in developing countries with practical suggestions to deal with problematic SVA use behaviors.

## 2 Literature review

### 2.1 Parasocial interaction and problematic SVA use

First introduced by Horton and Wohl (1956), parasocial interaction originally described the process in which television viewers foster imagined friendship or intimacy with media figures appearing in TV shows. Several factors contribute to the development of parasocial interaction in the TV setting, such as the characteristics of performers and the production techniques of programs (Rubin et al., 1985). Parasocial interaction has been investigated in various media environments, including social media and livestreaming platforms (Farivar et al., 2022; Hu et al., 2017).

Noticeably, compared with traditional media, SVA platforms provide users with a wide range of choices for sustaining parasocial interactions, such as viewing, liking, and commenting on vloggers' updates. While these behaviors may fail to capture vloggers' attention or facilitate two-way interpersonal interactions, users try to maintain the imagined closeness and intimacy with vloggers through their persistent engagement with short-form videos. Such a process is defined as parasocial interaction in the context of SVA, in which users forge a one-way connection with vloggers and imagine themselves engaging in a reciprocal relationship with them (Farivar et al., 2022; Horton and Wohl, 1956).

Previous research has shown that inappropriate interaction and engagement can result in negative outcomes such as problematic SVA use (Huang et al., 2022a; Wu-Ouyang, 2022). By continuously watching their favorite vloggers' posts, individuals develop a strong bond with vloggers and SVA, even without vloggers' feedback. This bond motivates users to invest increasing time and attention to stay tuned to vloggers' updates, which ultimately leads to problematic use behaviors. Drawing on prior definitions (Moretta et al., 2023; Wu-Ouyang, 2022), this study defines problematic SVA use as the compulsive engagement with SVA, the lack of self-control over such behaviors, and the associated adverse outcomes.

In recent years, due to the popularity of SVA, increasing studies have explored the phenomenon of problematic SVA use (Li et al., 2023; Yang et al., 2022). A growing body of studies have shown that individuals who engage in intense parasocial interactions with performers appearing in media content are more susceptible to using social media in a problematic manner (de Bérail et al., 2019; Farivar et al., 2022). Accordingly, we infer that users' frequent parasocial interactions with vloggers are likely to induce their problematic usage of SVA. More formally, this argument is formulated as the following hypothesis:

H1: In terms of the total effect, parasocial interaction is positively associated with problematic SVA use, without including any mediating variables.

### 2.2 Positive reinforcement approach: the mediating role of flow

From the psychological perspective, the positive reinforcement approach to explicating problematic use of digital tools emphasizes that the creation of positive experiences afforded by using online applications can foster users' dependence on these applications. In other words, the reinforcement of the positive feelings may ultimately result in maladaptive usage of online applications (Moretta et al., 2023; Gao et al., 2017). This approach has been supported in many empirical studies and typical predictors, such as flow, have been identified.

Flow is an optimal psychological experience in which individuals forget about other things, lose awareness of the passage of time, and feel a sense of self-transcendence and deep enjoyment (Csikszentmihalyi, 1991; Csikszentmihalyi and LeFevre, 1989). Likewise, this study defines flow as an immersive, concentrated, and joyful experience in which SVA users become fully absorbed in their parasocial interactions with vloggers. Therefore, based on the positive reinforcement approach, the following part tries to explicate the mediating role of flow in the association between parasocial interaction and problematic SVA use.

Parasocial interaction on SVA is characterized by users' imagination that they engage in a reciprocal relationship with vloggers, which indicates that this imagination is rarely interrupted by vloggers' performances and other external factors. This uninterrupted experience allows users to focus on their imagination for a long period, providing them with an immersive feeling and activating their flow experience. Individuals usually experience flow in a high-involvement environment (Bao and Yang, 2022; Cheng and Lu, 2015). Prior research has shown that users' flow experience emerges from their continuous use of online applications (Wu, 2009). Moreover, studies have supported a positive link between users' parasocial interaction with vloggers or live-streamers and flow (Hsu, 2020). Thus, we posit the following hypothesis:

H2.1: Parasocial interaction is positively associated with flow.

The brain's reward mechanism has implications for understanding the impact of flow on problematic use behaviors. When the brain receives an external stimulus, the secreted dopamine activates the reward circuit and enables individuals to experience intense pleasure and establish a connection between the stimulus and the reward (Clark and Zack, 2023). As a result, individuals often engage in repetitive behaviors to obtain a sense of reward, which subsequently induces their problematic use behaviors (Clark and Zack, 2023). For SVA users, the experience of flow functions as an intrinsic reward (Huang et al., 2022b). To maintain the flow experience, users spend more time in watching short-form videos, which ultimately causes problematic SVA use. Furthermore, previous literature has demonstrated that flow experienced by social media and smartphone users is positively associated with their problematic use behaviors (Qin et al., 2022; Wang, 2020). Therefore, we posit that a positive association exists between flow and the problematic usage of SVA:

H2.2: Flow is positively associated with problematic SVA use.

According to the positive reinforcement approach and based on H2.1 and H2.2, this study infers that flow may mediate the association between parasocial interaction and problematic SVA use. Thus, we put forward the following hypothesis:

H2.3: Flow mediates the association between parasocial interaction and problematic SVA use.

### 2.3 Compensatory use approach: the mediating role of fear of missing out

Unlike the positive reinforcement approach that sees positive experiences as a key predictor of problematic use behaviors, the compensatory use approach highlights the importance of negative feelings in inducing problematic use behaviors. Developed from the uses and gratifications theories, the compensatory use approach assumes that individuals use digital tools to regulate anxiety, loneliness, and stress, but the excessive use and inadequate self-regulation sometimes leads to problematic usage behaviors (Elhai et al., 2017; Wegmann and Brand, 2019). Many studies have supported the compensatory use approach and demonstrated that those who experience higher levels of negative emotions are more likely to engage in problematic use of online applications than those with lower levels of negative emotions (Hu et al., 2023; Mouakket and Aboelmaged, 2023; Zhang et al., 2022).

The fear of missing out is a common negative emotion that arises from an individual's strong need to build social connections with others (Cao et al., 2025; Gupta and Sharma, 2021). Drawing on the prior work (Brailovskaia et al., 2023), this study defines fear of missing out as a negative emotional state in which SVA users fear that they might miss out on vloggers' latest updates. Referring to the compensatory use approach (Elhai et al., 2017; Wegmann and Brand, 2019), the next paragraphs try to justify the mediating role of fear of missing in the association between parasocial interaction and problematic SVA use.

Similar to the dynamic of social interaction (Pimienta, 2023), when engaging in parasocial interactions on SVA platforms, users progressively develop familiarity and strong emotional connections with other users and media figures (Hsu, 2020; Zhang et al., 2019). However, faced with the endless stream of vloggers' updates and their language tactics, such as “don't miss other videos” or “see you in the next video,” users tend to establish an insecure attachment style where they worry that they cannot keep up with vloggers' pace and miss out on their latest updates (Schmuck, 2021). This process results in users' feeling of fear of missing out. Thus, the following hypothesis is posited:

H3.1: Parasocial interaction is positively associated with fear of missing out.

According to the compensatory motivation, users who experience negative emotions attempt to alleviate these feelings by spending increasing time using online applications (Servidio, 2023; Casale et al., 2024). For SVA users, frequently checking for their favorite vloggers' updates helps them manage their fear of missing out (Yang et al., 2022). Nevertheless, frequent checking and excessive use may eventually lead to problematic SVA use (Rahardjo and Mulyani, 2020). An increasing number of empirical studies have demonstrated a positive association between fear of missing out and problematic use of online applications (Griffiths and Kuss, 2017; Malaeb et al., 2021; Oberst et al., 2017). Formally, this association is articulated in the following hypothesis:

H3.2: Fear of missing out is positively associated with problematic SVA use.

The associations between the examined variables posited in H3.1 and H3.2 suggest a mediation model, in which fear of missing out mediates the association between parasocial interaction and problematic SVA use. This mediating mechanism aligns with the compensatory use approach, in which users attempt to manage their fear of missing out emerging from intense parasocial interactions with vloggers by frequently checking for updates. However, this maladaptive usage pattern leads to problematic use behaviors. Hence, the following hypothesis is proposed:

H3.3: Fear of missing out mediates the association between parasocial interaction and problematic SVA use.

### 2.4 Serial mediation effect of flow and fear of missing out

Some studies have employed flow and fear of missing out as separate conceptual tools to explicate the patterns of people's digital media use (Fauzi et al., 2021; Li et al., 2021). However, little is known about the association between these two concepts. Flow highlights the immersive enjoyment that users experience during their parasocial interactions with vloggers, while fear of missing out indicates users' attachment to the vloggers. The two concepts involve users' affinity for and their emotional dependence on vloggers, respectively. Although researchers argued for a positive link between fear of missing out and flow (Brailovskaia and Margraf, 2024), we propose that the direction for the link is opposite. Specifically, attachment theory offers insights into understanding the association between flow and fear of missing out (Li et al., 2021).

According to the attachment theory, individuals tend to seek intimacy and emotional connections with their attachment figures (Ren et al., 2012), especially those with an insecure attachment style. Vloggers act as attachment figures for SVA users (Ladhari et al., 2020). During the parasocial interactions, users' flow experience tends to increase their attachment to vloggers (Li and Peng, 2021), which subsequently intensifies their fear of missing out on vloggers' updates. To cope with this fear, users often compulsively scroll on SVA to ensure they have received the latest vlogs (Gupta and Sharma, 2021; Wang and Shang, 2024). Therefore, we infer that users' flow experience is likely to intensify their fear of missing out:

H4: Flow is positively associated with fear of missing out.

Taken together, these hypotheses suggest the serial mediating roles of flow and fear of missing out in the association between parasocial interaction and problematic SVA use. Given that flow and fear of missing out serve as key factors in the positive reinforcement and compensatory use approaches, respectively, this serial mediating path indicates that the creation and reinforcement of positive experiences may intensify the compensatory motivation for alleviating negative feelings in inducing users' problematic usage of SVA. Hence, the following hypothesis is proposed:

H5: Flow and fear of missing out serially mediate the association between parasocial interaction and problematic SVA use.

**Figure 1:**
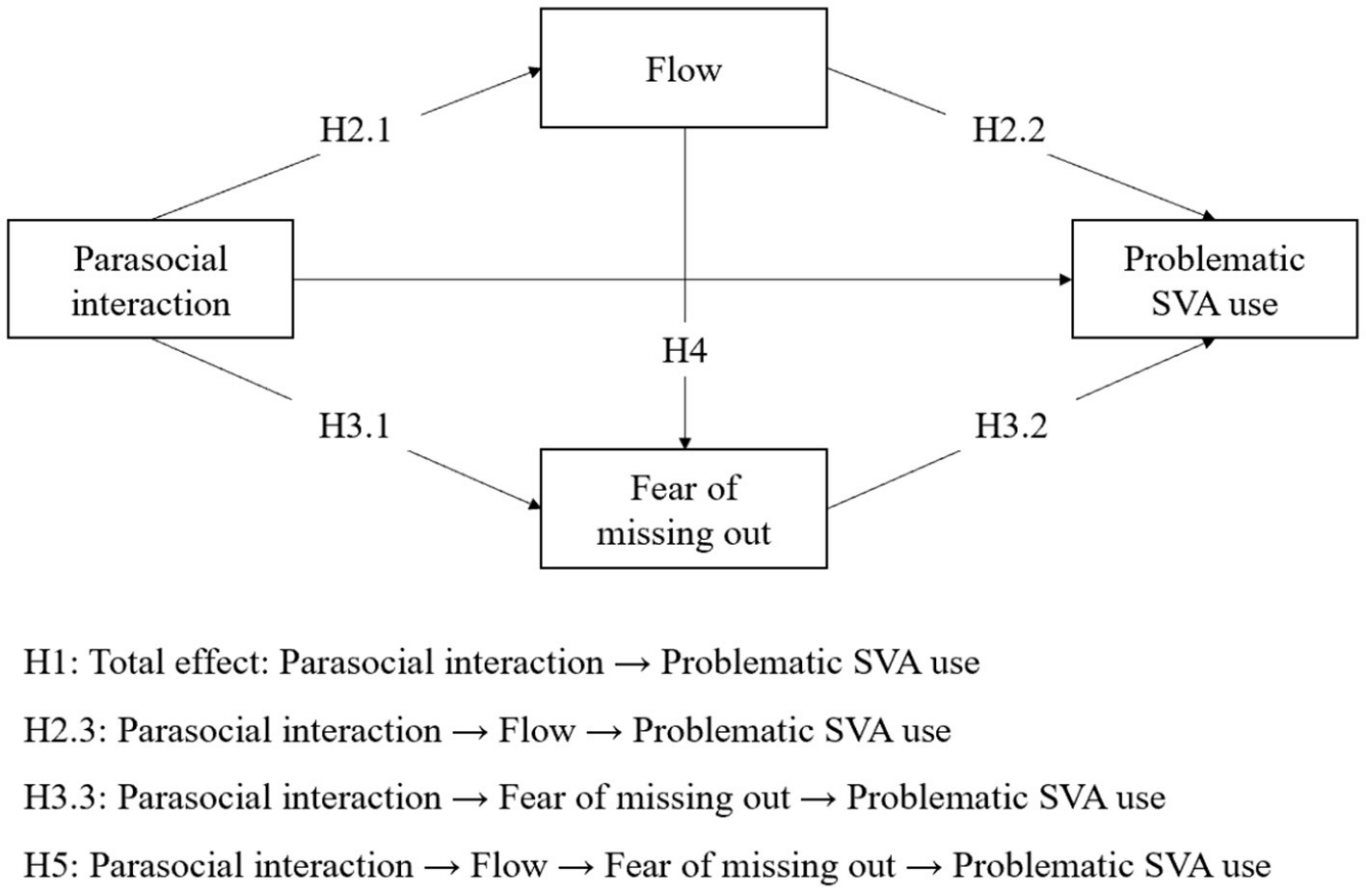
The hypothesized model.

Figure 1 illustrates the hypothesized model.

## 3 Methods

### 3.1 Participants and procedure

We used a cross-sectional online survey to collect data. The survey was conducted on Sojump, one of the biggest online survey platforms in China. Sojump has a sampling pool consisting of 6.2 million registered participants nationwide. According to the requested sample size, Sojump automatically generated random integers within the range of 1–6,200,000, which matched the respondents' ID numbers. Then, we sent emails to the chosen respondents and invited them to participate in the survey on a voluntary basis. This sampling strategy has been widely employed to investigate people's media use behaviors in general (Cui and Wu, 2021; Zhang and Fan, 2023) and to examine the problematic usage of SVA in particular (Feng et al., 2024).

The survey began on 11 July 2022 and ended on 17 July 2022. A total of 866 questionnaires were sent to users in Sojump's survey pool. We set screening questions (“Have you ever used SVA?”) to make sure that only participants with prior experience of using SVA were included in the sample. Four hundred twenty-three participants completed the questionnaire, achieving a response rate of 47.74%. In addition, participants who submitted the questionnaire multiple times or failed the attention checks were excluded. Moreover, 16 participants aged 15–17 were inadvertently included in the data collection process. Although we obtained informed consent directly from these minors, we were unable to contact their guardians due to the fully anonymized nature of the data. Upon identifying this issue, we reported it to the ethics review committee at the first author's affiliated institution. After thoroughly reviewing the questionnaire items, the committee concluded that the likelihood of harm to minor participants was extremely low, based on the following reasons: (1) All scales were adapted from peer-reviewed studies in both English and Chinese (Agarwat and Karahanna, 2000; de Bérail et al., 2019; Han et al., 2021; Huang et al., 2022a; Song et al., 2017; Wegmann et al., 2017; Wei et al., 2012) and were commonly employed in research with minors (Boer et al., 2020; Bond, 2016; Kaur et al., 2016; Oberst et al., 2017); (2) The questions focused exclusively on neutral topics such as media use and emotional states; and (3) No inquiries or complaints were received throughout the study. Therefore, we were advised to permanently delete all data from the 16 minor participants, including raw data, analytical files, and backups. Finally, 407 valid questionnaires were used for data analysis. At the significance level (α) of 0.05, this sample size outnumbered the recommended minimum of 179 observations calculated by G^*^Power (Faul et al., 2009; Xu and Tayyab, 2021). Table 1 presents the demographic characteristics of the respondents.

**Table 1:** Demographic characteristics of the participants (*N* = 407).

**Measure**	**Item**	**Frequency**	**Percentage (%)**
Gender	Male	166	40.8
	Female	241	59.2
Age	18–23	48	11.8
	24–30	188	46.2
	31–40	142	34.9
	41–45	14	3.4
	Over 45	15	3.7
Education Level	Never attended school	0	0
	Primary school	1	0.2
	Middle school	5	1.2
	High school	11	2.7
	Vocational high school	6	1.5
	Higher vocational school	31	7.6
	Bachelor	320	78.6
	Master	32	7.9
	Ph.D.	1	0.2
Monthly income	Less than RMB 1,500	20	4.9
	RMB 1,501–2,000	6	1.5
	RMB 2,001–3,000	20	4.9
	RMB 3,001–5,000	57	14.0
	RMB 5,001–8,000	117	28.7
	RMB 8,001–12,000	118	29.0
	RMB 12,001–20,000	51	12.5
	More than RMB 20,000	18	4.4
Duration of SVA use	< 30 min	24	5.9
	30 min to 1 h	82	20.1
	1–1.5 h	85	20.9
	1.5–2 h	88	21.6
	2–2.5 h	39	9.6
	2.5–3 h	36	8.8
	3–4 h	26	6.4
	4–5 h	17	4.2
	More than 5 h	10	2.5

### 3.2 Measures

#### 3.2.1 Parasocial interaction

Adapted from prior studies on the parasocial interaction between internet and social media users (de Bérail et al., 2019; Farivar et al., 2022), this study used a 14-item scale to measure SVA users' parasocial interaction with vloggers. Respondents were asked to suggest their agreement on a 5-point Likert scale ranging from 1 (“strongly disagree”) to 5 (“strongly agree”). Sample items included statements such as “My favorite vlogger makes me feel comfortable, as if I am with a friend,” “I look forward to watching my favorite vloggers in his or her next video,” and “I find my favorite vloggers attractive.” The items were averaged to create a composite index, where a higher score indicates a higher level of parasocial interaction (M = 3.86, SD = 0.48, Cronbach's α =0.84).

#### 3.2.2 Flow

Flow was measured with an established scale consisting of 13 items (Agarwat and Karahanna, 2000; Wang, 2020). Sample items included: when I imagine myself engaging in a reciprocal relationship with vloggers, (1) time seems to fly; (2) my attention remains concentrated; and (3) my curiosity is evoked. These items were asked on a 5-point Likert scale (1 = “strongly disagree,” 5 = “strongly agree”), with higher scores indicating higher levels of flow experience (M = 3.91, SD = 0.51, Cronbach's α = 0.86).

#### 3.2.3 Fear of missing out

According to previous research (Przybylski et al., 2013; Wegmann et al., 2017), the 6-item scale of fear of missing out was modified in the context of SVA usage. Sample items included: “I always stay online to make sure that I don't miss out on vloggers' updates,” “Commenting on the latest updates is important to me,” and “Understanding the language tactics used by vloggers is crucial to me.” These items were asked on a 5-point Likert scale (1 = “strongly disagree,” 5 = “strongly agree”), with higher values suggesting higher levels of fear of missing out (M = 2.98, SD = 0.81, Cronbach's α =0.87).

#### 3.2.4 Problematic SVA use

Drawing upon the previously used instrument (Huang et al., 2022a,b), problematic SVA use was measured using 6 items on a 5-point Likert scale (1 = “strongly disagree,” 5 = “strongly agree”). Sample items included: “I spend more time watching vloggers' videos than intended,” “I am often late for appointments due to watching vloggers' videos when I shouldn't be,” and “I find it difficult to disconnect from vloggers' videos.” A higher score represented a stronger tendency toward problematic SVA use (M = 2.63, SD = 0.85, Cronbach's α = 0.86).

#### 3.2.5 Control variables

This study included six control variables. A lack of self-control was included as a control variable and measured with 3 items on a 5-point scale (1 = “strongly disagree,” 5 = “strongly agree”). For example, one sample item was, “I often act without thinking carefully about all the alternatives” (M = 2.83, SD = 0.90, Cronbach's α = 0.80). Considering that the duration of media use is a predictor of problematic use (Zhang and Rau, 2021), duration of SVA usage was entered as another control variable. It was measured with a single item: “On average, how much time do you spend watching vloggers' videos every day?” (median = 4.00 or 1.5–2 h, SD = 1.97). Moreover, gender was measured as a dichotomous variable (40.4% males) and age as a continuous variable (M = 30.31, SD = 6.62). Education level (median = 7.00 or bachelor's degree, SD = 0.84) and monthly income (median = 5.00 or RMB 5,001–8,000, SD = 1.55) were measured as ordinal variables. The full text of the questionnaire is provided in the Appendix.

### 3.3 Analytic strategy

Amos 28.0, SPSS version 26.0 and PROCESS version 3.5 were used to analyze the data. We first used Amos 28.0 to conduct confirmatory factor analyses (CFAs) to evaluate construction and validity of the instruments. Then, using the SPSS 26.0 program, we calculated the means and standard deviations of the examined variables and the bivariate correlations between them. Next, we employed Model 6 of the PROCESS macro (Hayes, 2013) to test the serial mediation effect, using 5,000 bootstrap samples at 95% bias-corrected confidence intervals. Problematic SVA use was entered as the dependent variable and parasocial interaction as the independent variable. Flow and fear of missing out were entered as mediators. Lack of self-control, gender, age, education level, and monthly income were entered as covariates. Standardized coefficients were reported.

## 4 Data analysis and results

### 4.1 Statistical assumption tests

The results of the CFAs indicated that the model fit for parasocial interaction was acceptable, with χ^2^/df = 3.585 < 5, RMR = 0.038 < 0.08, GFI = 0.908 > 0.90. The model fit for fear of missing out was also acceptable, with χ^2^/df = 4.570 < 5, RMR = 0.035 < 0.08, GFI = 0.966 > 0.90. For the initial models of flow and problematic SVA use, some fit indices fell below standard thresholds. Subsequently, based on the modification indices (MI), minor adjustments were made to the residuals. After revision, the fit indices of flow (χ^2^/df = 3.950 < 5, RMR = 0.041 < 0.08, GF I = 0.905 > 0.90) and problematic SVA use (χ^2^/df = 4.530 < 5, RMR = 0.048 < 0.10, GFI = 0.972 > 0.90) improved to acceptable levels. Moreover, Cronbach's α coefficients for all constructs exceed 0.70. Thus, the measurements in this study are statistically valid and reliable.

Variance inflated factor (VIF) and tolerance value were calculated to test the multi-collinearity. The results showed that all VIFs were lower than 10 and all tolerance values were above 0.10, indicating that there was no co-linearity problem. Harman's single-factor analysis was performed to test the common method bias. The results showed that there were nine factors with eigenvalues >1, and the first factor accounted for 24.79% of the variance, which was less than the critical criterion of 40%. This suggested no substantial common method bias in this study (Podsakoff and Organ, 1986). Moreover, we tested the correlation coefficients among the examined variables. The findings suggested a minimal likelihood of common method bias, because there was no unexpectedly high correlation (>0.9) within the correlation matrix (Pavlou et al., 2007). Table 2 presents the means, standard deviations, and bivariate correlations between the examined variables.

**Table 2:** Means, standard deviations, and bivariate correlations between examined variables.

**Variables**	**M**	**SD**	**1**	**2**	**3**	**4**	**5**	**6**	**7**	**8**	**9**	**10**
1. Parasocial interaction	3.86	0.48	1									
2. Flow	3.91	0.51	0.70^***^	1								
3. Fear of missing out	2.98	0.81	0.58^***^	0.56^***^	1							
4. Problematic SVA use	2.63	0.85	0.06	0.26^***^	0.22^***^	1						
5. Lack of self-control	2.83	0.90	−0.18^***^	0.06	−0.03	0.61^***^	1					
6. Duration of SVA use			0.18^***^	0.18^***^	0.21^**^	0.18^***^	0.10^*^	1				
7. Gender	–	–	−0.11^*^	−0.11^*^	−0.10	0.12^*^	0.07	0.03	1			
8. Age	30.31	6.62	0.17^***^	0.15^**^	0.15^**^	−0.09	−0.18^***^	−0.15^**^	−0.12^*^	1		
9. Education level	7.00 (median)	0.84	0.11^*^	0.07	−0.02	0.07	0.06	−0.01	−0.002	−0.12^*^	1	
10. Monthly income	5.00 (median)	1.55	0.28^***^	0.14^**^	0.17^**^	−0.12^*^	−0.17^***^	0.05	−0.18^***^	0.31^***^	0.30^***^	1

Gender 1 = male, 2 = female; ^*^p < 0.05; ^**^p < 0.01; ^***^p < 0.001.

### 4.2 Examining mediation effects

We ran a serial mediation analysis to test the hypotheses. Consistent with H1, the association between parasocial interaction and problematic SVA use was significant without including any mediators (β = 0.17, *p* < 0.001). However, after including two mediating variables, namely flow and fear of missing out, the direct association between parasocial interaction and problematic SVA use was not significant (β = −0.05, *p* = 0.42). In line with H2.1 and H2.2, parasocial interaction was positively associated with flow (β = 0.73, *p* < 0.001), and flow was positively correlated to problematic SVA use (β = 0.16, *p* < 0.01). Moreover, bootstrap tests with 5,000 samples were performed to examine the indirect associations between parasocial interaction and problematic SVA use via flow and fear of missing out. The indirect association was significant if the 95% confidence intervals did not include 0. The results demonstrated that flow significantly and positively mediated the association between parasocial interaction and problematic SVA use [β = 0.12, CI_95%_ [0.03, 0.20]], which supported H2.3.

Supporting H3.1 and H3.2, a positive association was identified between parasocial interaction and fear of missing out (β = 0.35, *p* < 0.001), with the latter demonstrating a positive association with problematic SVA use (β = 0.18, *p* < 0.001). According to the bootstrap test, the indirect association between parasocial interaction and problematic SVA use through fear of missing out was significant [β = 0.06, CI_95%_ [0.03, 0.10]], showing support for H3.3.

In addition, flow was positively associated with fear of missing out (β = 0.29, *p* < 0.001), supporting H4. Finally, consistent with H5, flow and fear of missing out serially mediated the association between parasocial interaction and problematic SVA use [β = 0.04, CI_95%_ [0.02, 0.06]].

**Figure 2:**
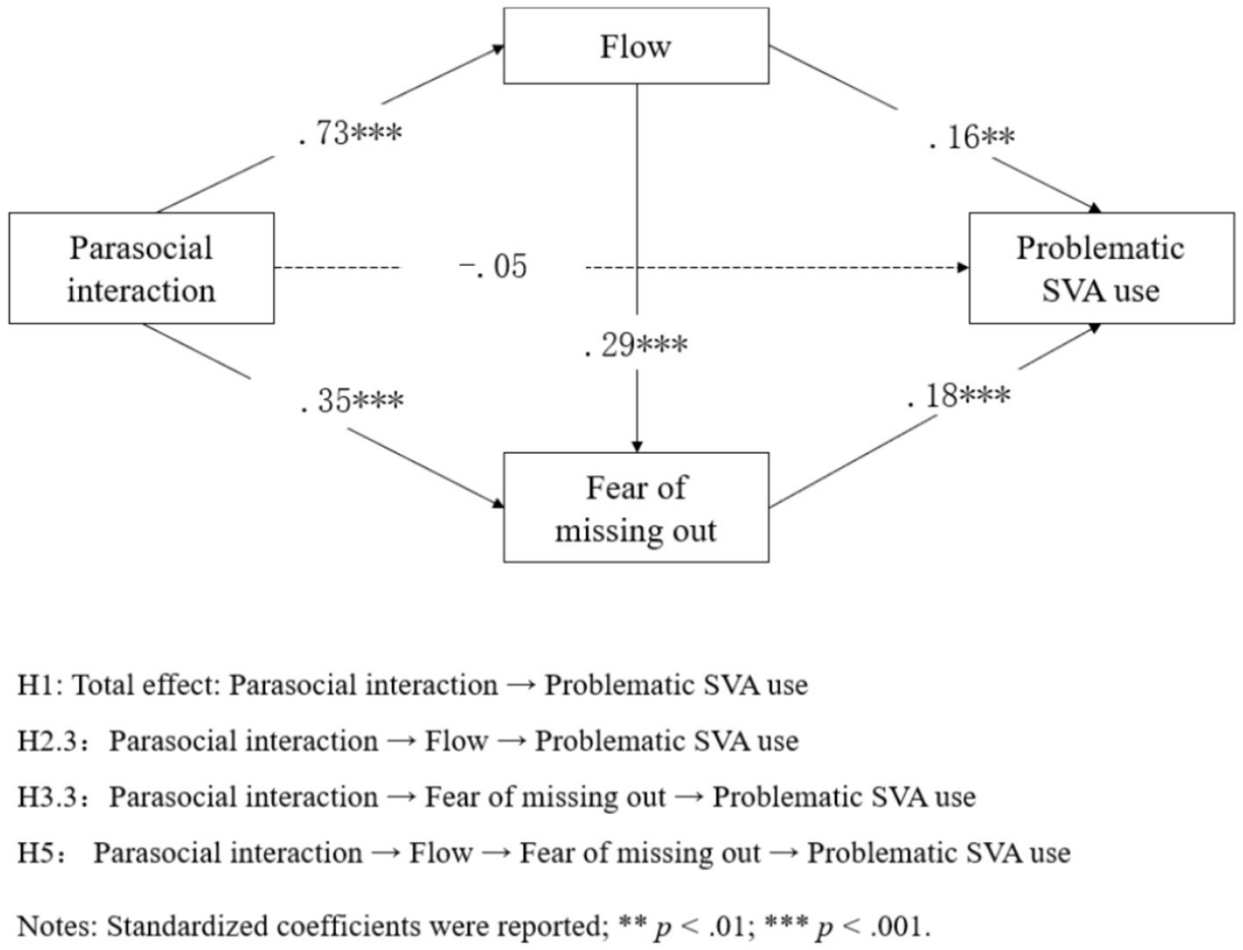
The final model based on the statistical results. Standardized coefficients were reported; **p < 0.01; ***p < 0.001.

Table 3, Figure 2 illustrate the results.

**Table 3:** Results of indirect associations.

**Paths**	**Standardized (β)**	**95% CI**	
		**Low**	**High**
Parasocial interaction → flow → problematic SVA use	0.12	0.03	0.20
Parasocial interaction → fear of missing out → problematic SVA use	0.06	0.03	0.10
Parasocial interaction → flow → fear of missing out → problematic SVA use	0.04	0.02	0.06

SVA, short-form video applications.

## 5 Discussion

### 5.1 Key findings

Considering users' parasocial interactions with vloggers a common type of socio-psychological process in the context of SVA usage, this study attempts to examine how parasocial interaction induces problematic SVA use through flow and fear of missing out. Several key findings are drawn from the present study.

First, parasocial interaction was positively associated with problematic SVA use when no mediators were included. Through parasocial interactions, which highlights users' imagination of engaging in a reciprocal relationship with vloggers (Garg and Bakshi, 2024), users built an emotional bond with vloggers. This connection motivated users to spend an excessive amount of time staying tuned to the latest updates, thereby leading to the side effect of problematic SVA use. The finding is in line with attachment theory that users' attachment to online members may contribute to their addictive use of SVA (Zhang et al., 2019). However, after the inclusion of flow and fear of missing out as mediators in the model, the association between parasocial interaction and problematic SVA use became insignificant. In other words, although users' parasocial interactions with vloggers tended to induce problematic SVA use, flow and fear of missing out were the proximal predictors of problematic use behavior. Accordingly, users' parasocial interactions with vloggers was an antecedent to problematic SVA use: it first activated users' flow experience and fear of missing out, which in turn led to problematic use behaviors.

Second, flow fully mediated the association between parasocial interaction and problematic SVA use. By fully engaging in the parasocial interaction with vloggers, SVA users experienced flow, characterized by an optimal feeling of deep immersion and self-transcendence (Csikszentmihalyi and LeFevre, 1989), which then induced their problematic use behaviors. These findings corroborate the positive reinforcement approach (Gao et al., 2017; Qin et al., 2022) and emphasize that the creation and reinforcement of positive experiences like flow plays a crucial role in developing problematic SVA use. While previous work mainly focused on the positive aspects of flow experience, the findings demonstrate its negative impact, that is, inducing problematic SVA use behaviors.

Third, fear of missing out fully mediated the association between parasocial interaction and problematic SVA use. Frequent parasocial interactions with vloggers often activated users' desire to establish an emotional connection with vloggers (Schmuck, 2021). However, users' imagination of participating in a reciprocal relationship with vloggers (Farivar et al., 2022; Horton and Wohl, 1956), instead of engaging in a real-time and two-way interaction, might not satisfy this need. Hence, the failure to meet the users' need triggered a sense of fear of missing out among them. Meanwhile, users tried to manage this fear by compulsively checking for vloggers' updates, which finally led to problematic use behaviors (see Elhai et al., 2017; Griffiths and Kuss, 2017; Oberst et al., 2017). These findings are in line with the compensatory use approach (Elhai et al., 2017; Kardefelt-Winther, 2014) and highlight the role of compensatory motivation for alleviating negative emotions in inducing problematic SVA use.

Fourth, flow and fear of missing out serially mediated the association between parasocial interaction and problematic SVA use. Put differently, during users' parasocial interactions with vloggers, it was flow experience that intensified fear of missing out, which subsequently led to problematic SVA use. These findings suggest that the positive reinforcement approach plays a more fundamental role than the compensatory use approach does in explicating the mechanism underlying the association between parasocial interaction and problematic use of SVA.

### 5.2 Theoretical implications

Overall, our study addresses the first and second goals outlined in the introduction section. By conceptualizing users' parasocial interactions with vloggers as a socio-psychological process on the SVA platforms, we explain the mediating mechanism underlying parasocial interaction and problematic SVA use. Specifically, the theoretical implications are discussed as follows.

First, the association between parasocial interaction and problematic SVA use delineates the socio-psychological mechanism of the formation of problematic use behaviors. Prior research mainly employed a psychological perspective to explicate problematic use of online applications (Zhang and Rau, 2021), whereas users' interactions with vloggers were ignored. To fill in the research gap, this study introduces parasocial interaction as a common type of socio-psychological process in the setting of SVA use and treats it as an antecedent that explains the development of problematic SVA use, thereby incorporating the social factor into the psychological perspective in problematic behaviors research.

Second, the mediating role of flow and fear of missing out in the association between parasocial interaction and problematic SVA use helps clarify the psychological approaches. Previous studies tended to consider users' parasocial interactions with vloggers or influencers on social networking sites (SNS) a direct cause of problematic use of SNS (de Bérail et al., 2019; Farivar et al., 2022). Nevertheless, the mechanism underlying this association has received little attention. By drawing on the positive reinforcement and compensatory use approaches to include flow and fear of missing out as mediators, the findings elucidate the psychological mechanisms underlying parasocial interaction and problematic SVA use.

Third, the serial mediation effect of flow and fear of missing out integrates the positive reinforcement approach with the compensatory use approach, further refining the psychological mechanism underlying parasocial interaction and problematic SVA use. That is, the creation and reinforcement of positive experiences tend to intensify the compensatory motivation for alleviating negative emotions in forming problematic SVA use. Prior studies either treated flow and fear of missing out as separate predictors of digital media use patterns (Fauzi et al., 2021; Li et al., 2021), or argued that fear of missing out predicted flow (Brailovskaia and Margraf, 2024). In response, our study offers an alternative explanation for how flow and fear of missing out interact within the psychological mechanism that results in problematic SVA use. Moreover, the serial mediation effect suggests that disrupting the loop of reinforcing positive feelings may reduce the risk of developing problematic SVA use.

### 5.3 Practical implications

The findings have several practical implications for preventing users from falling into problematic use behaviors, which addresses the third goal of this study. First, considering that flow mediated the association between parasocial interaction and problematic SVA use, SVA platforms could execute pop-up notifications to remind users about their usage time. Through such interruptions, temporal dissociation—a crucial element of flow—is disrupted, thereby reducing the likelihood of problematic SVA use (Huang et al., 2022a).

Second, given the mediating role of fear of missing out in the association between parasocial interaction and problematic SVA use, platforms should improve their algorithms and recommend a wider range of videos to users. Parasocial interaction with diverse types of vloggers may help lower users' attachment to certain vloggers and alleviate their worry about missing out the updates. This, in turn, could lower the possibility of forming problematic use behaviors.

Third, the indirect associations of parasocial interaction on problematic SVA use suggest that users strike a balance between digitally mediated parasocial interaction and face-to-face interaction. Heavy SVA users are encouraged to cultivate skills and strategies for disconnecting from these applications. For instance, turning off notifications and adjusting privacy settings may help digital disconnection. Such practices provide SVA users with more time to take part in in-person conversations and increase subjective wellbeing (Nguyen et al., 2024). These steps serve as effective strategies to prevent users from developing problematic SVA use.

### 5.4 Limitations and future research

This study has several limitations that warrant further exploration. First, although our sample was closely representative of SVA users in China regarding gender and age distribution,^2^ other demographic characteristics of our participants, please refer to Table 1. other demographic characteristics like education and income were not adequately represented. Therefore, readers should cautiously generalize the findings to the population. To increase the generalizability of the findings, future studies should use quota sampling or stratified sampling that considers the ratios of gender, age, education level, income, and area of residence of the population to collect data.

Second, although the positive reinforcement and compensatory use approaches theoretically supported the proposed model in this study, causality cannot be inferred due to the cross-sectional nature of the data. In other words, it is possible that problematic SVA users are more likely to engage in parasocial interactions with vloggers compared with those with a lower level of problematic SVA use. Longitudinal surveys or experiments are needed to test the causal relationships between the examined variables in the future.

Third, despite the indirect associations of parasocial interaction on problematic SVA use through flow, through fear of missing out, and through both mediators were all significant, no statistically significant differences were found between them. This indicates that if some moderating variables had been included, the differences between the indirect associations might have become significant. Thus, future research could enlarge the sample size and identify the possible moderator that divides the sample into several subsamples. By testing the proposed mediation model across the distinct groups, researchers may observe the difference between the positive reinforcement and compensatory use approaches in understanding problematic SVA use.

## 6 Conclusion

In the era of deep mediatization (Couldry and Hepp, 2013; Livingstone, 2009), smartphones and mobile applications are increasingly penetrating into every aspect of our everyday life, reshaping modes of thinking and behavior. Examining the problematic use of SVA, one of the most popular mobile applications in China (Wang and Scherr, 2022), has great theoretical and practical significance for understanding individuals' psychological wellbeing. By proposing flow and fear of missing out as key mediators between parasocial interaction and problematic SVA use, our model illuminates the dual roles of positive emotional reinforcement and compensatory motivation of mitigating negative emotions in driving problematic behaviors. These findings enhance our understanding of the socio-psychological processes underlying the problematic usage of SVA and other digital media. Moreover, the study suggest that users break free from isolating digital bubbles (Turkle, 2012) and foster genuine social connections to lower the risk of developing maladaptive usage of digital media.

## Data Availability

The raw data supporting the conclusions of this article will be made available by the authors, without undue reservation.

## Footnotes

^1^Generally, vloggers are content creators who produce short-form videos to attract followers. Vloggers consist of two types: (1) those who create and perform in the videos; and (2) those who only produce videos but do not perform in videos. This study focuses on the former type.

^2^By the first quarter of 2022, males accounted for about 45% of SVA users in China, while the remaining 55% were females. In terms of age, 22% of SVA users were between 18 and 23 years old, 49% were between 24 and 30 years old, and 21% were between 31 and 40 years old. Data were retrieved from https://www.thepaper.cn/newsDetail_forward_18710654.
