# Supplementary material for: Parasocial interaction and problematic use of short-form video applications: unveiling the mediating mechanism
Source: Front Psychol. 2025 Jul 14;16:1584685. doi: 10.3389/fpsyg.2025.1584685 (PMC12301365; doi:10.3389/fpsyg.2025.1584685)
Supplement: Supplementary file 1 [file Presentation_1.pdf]

## Appendix: Variables and Scales

### Parasocial Interaction

To what extent do you agree with the following statements:

1. My favorite vlogger makes me feel comfortable, as if I am with a friend;
2. I see my favorite vlogger as a natural, down-to-earth person;
3. I look forward to watching my favorite vloggers in his or her next video;
4. My favorite vloggers seem to understand the kinds of things I want to know;
5. I find my favorite vloggers attractive;
6. I can trust the information provided to me by these vloggers;
7. I think the information provided by these vloggers is in line with my interests;
8. I miss seeing my favorite vlogger when he or she is ill or on vacation;
9. I feel sorry for my favorite vlogger when he or she makes a mistake;
10. If my favorite vloggers don't release any new videos, I will have a sense of loss;
11. Watching their vlogs makes me feel very happy;
12. I would like to meet my favorite vloggers in person;
13. If my favorite vlogger recommends a product, I will buy it;
14. If I come across these vloggers in newspapers, magazines, on the Internet or other media, I will read this report.

### Flow

To what extent do you agree with the following statements:

1. While watching videos from my favorite vloggers, time seems to fly;
2. Sometimes I lose track of time when I am watching videos from my favorite vloggers;
3. Most times when I am watching these vlogs, I end up spending more time that I had planned;
4. While watching videos from my favorite vloggers, I am able to block out most other distractions;
5. While watching videos from my favorite vloggers, I am absorbed in what I am doing;
6. While watching videos from my favorite vloggers, I am immersed in the task I am performing;
7. While watching videos from my favorite vloggers, my attention does not get diverted very easily;
8. I have fun interacting with these vloggers;
9. Watching videos from these vloggers provides me with a lot of enjoyment;
10.I enjoy watching videos from my favorite vloggers;
11. Watching videos from my favorite vloggers excites my curiosity;
12. Interacting with my favorite vloggers makes me curious;
13. Watching videos from my favorite vloggers arouses my imagination.

### Fear of Missing Out

To what extent do you agree with the following statements:

1. I always stay online to make sure that I don’t miss out on my favorite vloggers’ updates;
2. Commenting on these latest updates is important to me;
3. I fear not to be up-to-date in following the latest updates from my favorite vloggers;
4. I continuously scroll my short-form video app, in order not to miss out on information from my favorite vloggers;
5. Understanding the Internet buzzwords used by these vloggers is crucial to me;

6. When I go on vacations, I continue to keep tabs on what my favorite vloggers are doing.

## Problematic Use

To what extent do you agree with the following statements:

1. I have difficulties in focusing on my study or work due to scrolling short-form videos;
2. I spend more time scrolling short-form videos than intended, which led to my lack of sleep;
3. I am often late for appointments due to scrolling short-form videos when I shouldn’t be;
4. My family or friends think that I spend too much time on scrolling short-form videos;
5. I feel anxious if I cannot access to short-form videos;
6. I find it difficult to disconnect from short-form videos.
